# High visceral fat attenuation and long‐term mortality in a health check‐up population

**DOI:** 10.1002/jcsm.13226

**Published:** 2023-04-05

**Authors:** Jong Hyuk Lee, Seung Ho Choi, Keum Ji Jung, Jin Mo Goo, Soon Ho Yoon

**Affiliations:** ^1^ Department of Radiology Seoul National University College of Medicine Seoul Korea; ^2^ Department of Internal Medicine, Healthcare Research Institute, Healthcare System Gangnam Center Seoul National University Hospital Seoul Korea; ^3^ Department of Epidemiology and Health Promotion, Institute for Health Promotion, Graduate School of Public Health Yonsei University Seoul Korea; ^4^ Institute of Radiation Medicine Seoul National University Medical Research Center Seoul Korea; ^5^ Cancer Research Institute Seoul National University Seoul Korea

**Keywords:** body composition, deep learning, prognosis, sarcopenia, survival, visceral fat

## Abstract

**Background:**

The prognostic role of increased visceral fat attenuation (VFA) remains underexplored. We investigated the long‐term prognostic implications of computed tomography (CT)‐derived VFA in a health check‐up population.

**Methods:**

This study included consecutive individuals who had positron‐emission tomography/CT scans for health check‐ups between January 2004 and December 2010. The primary outcome was overall survival (OS), and the secondary outcomes were cancer‐specific survival (CSS) and non‐cancer‐specific survival (NCS). Commercially available body composition analysis software was used to obtain abdominal waist VFA, visceral fat volume index (VFI) and skeletal muscle index (SMI) at the L3 level. Sarcopenia was determined using sex‐specific SMI references. VFA and VFI were dichotomized using the thresholds for the highest quartiles. The relationship between CT‐derived body composition parameters and body mass index (BMI) was evaluated with Pearson correlation coefficients. The prognostic implications of VFA and sarcopenic obesity (SO) defined by VFA were assessed by multivariable Cox regression analysis and Kaplan–Meier plots with log‐rank tests.

**Results:**

A total of 2720 individuals (1530 men [56.3%] and 1190 women [43.7%]; median age: 53 years, inter‐quartile range: 47–60 years) were included. During the median follow‐up of 138 months, 128 individuals (5%) died (cancer mortality: 2%; non‐cancer mortality: 3%), with 0.2% (5 of 2720) and 1.1% (30 of 2720) of 1‐ and 5‐year mortality rates. VFA was negatively correlated with BMI (*r* = −0.62; *P* < 0.001) and VFI (*r* = −0.69; *P* < 0.001). After adjusting for clinical variables, sarcopenia and VFI, high VFA was a negative prognostic factor for OS (hazard ratio [HR]: 1.05 per Hounsfield unit; 95% confidence interval [CI]: 1.02, 1.08; *P* = 0.001), CSS (HR: 1.07 per Hounsfield unit; 95% CI: 1.02, 1.12; *P* = 0.006) and NCS (HR: 1.03 per Hounsfield unit; 95% CI: 1.01, 1.06; *P* = 0.009). Individuals with high VFA had higher high‐sensitivity C‐reactive protein levels than those with low VFA (0.11 vs. 0.03 mg/dL; *P* < 0.001). Individuals with SO defined by VFA had worse OS (9% vs. 4%; *P* < 0.001), CSS (3% vs. 2%; *P* = 0.02) and NCS (6% vs. 3%; *P* < 0.001) than those without SO, even in the same BMI (underweight‐to‐normal BMI, OS: 8% vs. 4%; overweight‐to‐obese BMI, OS: 38% vs. 4%; *P* < 0.001 in both) or VFI category (high VFI, OS: 43% vs. 6%; low VFI, OS: 8% vs. 3%; *P* < 0.001 in both).

**Conclusions:**

High VFA was associated with long‐term mortality and low‐grade inflammation. VFA can further stratify the current SO by BMI or VFI, and SO defined by VFA can identify individuals who are most vulnerable to long‐term mortality.

## Introduction

Obesity is defined as abnormal or excessive fat accumulation that poses a risk to health.^1^ It is a risk factor for various chronic diseases, including cancer, cardiovascular disease, diabetes mellitus (DM) and chronic kidney disease, leading to early morbidity and mortality.^1^, ^2^ The prevalence of obesity has explosively increased since 1975, and it is now one of the most severe global public health problems, as 13% of adults worldwide have obesity as of 2016.^1^, ^2^

Body mass index (BMI), which is calculated from height and weight, is a surrogate to diagnose obesity.^1^, ^2^, ^3^ However, despite its ease of use, BMI is only a measure of weight (i.e., a sum of body fat, muscle, bone and organs), not body fat alone.^1^, ^2^, ^4^ Another problem is that other factors established to influence obesity, such as sex, age and race, cannot be considered in BMI. To overcome these limitations, direct indexes for body fat measurements from computed tomography (CT) images, focusing on fat depots, volume, density and their interactions, have been investigated.^5^, ^6^, ^7^, ^8^, ^9^, ^10^, ^11^ Indeed, visceral adiposity has been reported to be associated with poor overall and cardiovascular mortality.^5^, ^6^, ^7^, ^8^, ^9^, ^10^, ^11^

Although recent studies reported that visceral fat attenuation (VFA) plays a vital role as a biomarker of cardiovascular diseases, metabolic syndrome and mortality, conflicting evidence has been reported regarding the prognostic role of VFA.^5^, ^6^, ^8^, ^10^, ^12^ Specifically, although low VFA has been reported to be positively associated with metabolic syndrome, including cardiovascular risk,^6^, ^8^ high VFA has been identified as a predictor of mortality.^5^, ^10^ In addition, considerable heterogeneity exists in the measurement location for VFA, such as at a single slice or specific level of the lumbar vertebrae (e.g., the L4–L5 disc space) in prior studies.^5^, ^6^, ^8^, ^10^, ^12^ However, because the visceral fat distribution differs craniocaudally in the abdomen, a volumetric analysis fully capturing all fat in the entire region would be more accurate.^13^ Therefore, this study investigated the long‐term prognostic implications of CT‐derived VFA using volumetric analysis in a health check‐up population.

## Methods

This retrospective study was approved by the Institutional Review Board (IRB) of Seoul National University Hospital, and the requirement for written informed consent was waived (IRB No. H‐2010‐122‐1166). The study population was not reported before.

### Study population and data collection

This study was performed at a single medical check‐up centre (Seoul National University Hospital Healthcare System Gangnam Center, Seoul, Korea), which provides a comprehensive medical check‐up programme for non‐communicable diseases.^14^ Positron‐emission tomography (PET)/CT examinations were performed as one of the check‐up examinations when participants wanted cancer screening without any symptoms or signs.^15^ This type of medical check‐up, in which participants pay for the screening costs at their own expense, is common in Northeast Asia.^16^, ^17^

**Figure 1:**
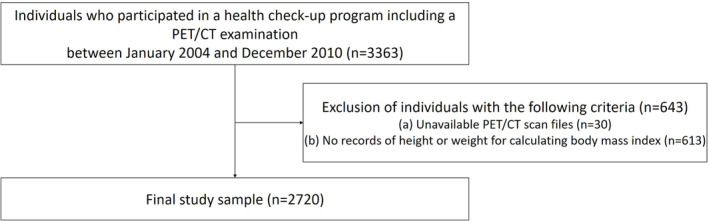
Flow diagram for this study. CT, computed tomography; PET, positron‐emission tomography.

All participants who underwent PET/CT between January 2004 and December 2010 were consecutively collected. We included the first PET/CT scan if individuals had multiple PET/CT examinations. The exclusion criteria were as follows: (a) individuals without available PET/CT scan files (*n* = 30) and (b) individuals without records of height and weight at the time of PET/CT scans (*n* = 613) (*Figure* *1*).

The following clinical data were obtained from individuals' electronic medical records and self‐reported questionnaires: demographic information (age, sex, height, weight and BMI), smoking status (never, former and current smoking), previous disease history (previous cancer history, hypertension, DM, cardiovascular disease, cerebrovascular disease, chronic liver disease and chronic renal disease) and high‐sensitivity C‐reactive protein (hs‐CRP) level obtained at the same day of the PET/CT examination. BMI was categorized as underweight (<18.5 kg/m^2^), normal (18.5–24.9 kg/m^2^), overweight (25–29.9 kg/m^2^) and obese (>30 kg/m^2^).^2^

### Image acquisition and body composition analysis

^18^F‐Fluorodeoxyglucose PET/CT scans were obtained with one scanner (Gemini Dual GS, Philips). Non‐contrast torso CT images were acquired from the skull base to the mid‐thigh with the following parameters: section thickness, 6.5 mm; section interval, 6.5 mm; tube voltage, 140 kVp; and tube current, 500 mAs.

Non‐contrast torso CT images were imported into a commercially available deep learning‐based body composition analysis software (DeepCatch, v1.1.8.0, MEDICALIP Co., Ltd.).^S1–S3^ Two authors (J.H.L. and S.H.Y. with 10 and 17 years of experience in body images) confirmed the completeness of the segmentation of the software. The software calculated CT‐derived parameters, including total fat volume (cm^3^), visceral fat volume (cm^3^), subcutaneous fat volume (cm^3^), VFA (Hounsfield units [HU]) and subcutaneous fat attenuation (SFA; HU) at the abdominal waist level (World Health Organization definition; between the 12th rib and iliac crest) and skeletal muscle area at L3 (cm^2^).^18^ Further detailed information about the software has been described in a previous study.^13^

The total, visceral and subcutaneous fat volumes and skeletal muscle area were normalized for height in square metres to calculate the total fat volume index, visceral fat volume index (VFI), subcutaneous fat volume index (SFI) and skeletal muscle index (SMI). The cutoff value of SMI for sarcopenia was defined as 55 cm^2^/m^2^ for males and 39 cm^2^/m^2^ for females.^19^ Because there are no established cutoff values of the fat volume indexes and fat attenuation for survival outcomes, we arbitrarily split them into the highest quartile and others for analyses using categorical values.

### Outcomes

The primary outcome of this study was overall survival (OS), defined as the period from the date of individuals' PET/CT examination to the date of death from any cause. Survival time was censored on 31 December 2018. For individuals who died, the time of censoring was defined as the date of death. The secondary outcome was cancer‐specific survival (CSS) and non‐cancer‐specific survival (NCS), measured from the date of individuals' first PET/CT examination to the death from cancer or non‐cancer cause, respectively. For individuals who died from cancer or non‐cancer causes, the time of censoring was defined as the time of death from those causes. Survival status and date and cause of death were acquired from a database of the Statistics Korea.

### Statistical analysis

Baseline characteristics were compared between individuals who died and survived in the follow‐up period with Student's *t* test for continuous variables and the Pearson chi‐squared test for categorical variables. Pearson correlation coefficients were used to establish the relationship between CT‐derived body composition parameters and BMI. We also investigated the relationship between VFA and hs‐CRP levels by the Mann–Whitney *U* test according to the results obtained for the normality of the data distribution.

Univariable and multivariable Cox regression analyses were performed to evaluate the prognostic implications of VFA for OS, CSS and NCS. Multivariable Cox regression analyses were performed with backward stepwise selection, using variables with a *P* value <0.2 in the univariate analysis. Backward stepwise selection was conducted with an iterative entry of variables based on the test results (*P* < 0.05), and variables were removed based on likelihood ratio statistics with a probability of 0.1. To derive robustness, we separately performed the Cox regression analyses with continuous and categorical variables of CT‐derived parameters as input variables. Because ~24% of the study population (652 of 2720) had missing data for their smoking status, a complete case analysis was performed, followed by multiple imputations performed using the fully conditional specification method. Five imputed data sets were generated.

To investigate the prognostic value of sarcopenic obesity (SO) defined by BMI, VFI, VFA or both VFI and VFA, we calculated the *C* index using Uno's concordance statistics.^S4,S5^ Kaplan–Meier plots with log‐rank tests were performed according to whether or not individuals had SO defined by VFA, even in the same BMI or VFI category. As a sensitivity analysis, we performed the log‐rank test to confirm the prognostic implications of SO‐defined visceral fat abnormality (the highest quartile of VFA or VFI).

All statistical analyses were performed using SPSS Version 21.0 (IBM Corp.), SAS Version 9.4 (SAS Institute Inc.) and R Version 3.6.1 (R Project for Statistical Computing), and a *P* value of <0.05 was considered to indicate statistical significance.

## Results

### Baseline characteristics

A total of 2720 individuals (1530 men [56.3%] and 1190 women [43.7%]; median age: 53 years, inter‐quartile range [IQR]: 47–60 years) were included in this study. The baseline characteristics of this study population are described in *Table* *1*. In a total follow‐up duration of 138 months (IQR: 120–153 months), 128 individuals (5%) died, with cancer mortality in 2% (47 of 2720) and non‐cancer mortality in 3% (81 of 2720). The 1‐ and 5‐year mortality rates were 0.2% (5 of 2720) and 1.1% (30 of 2720), respectively.

**Table 1:** Baseline characteristics of the study population

Clinical variables	Study population (*n* = 2720)	Individuals who died (*n* = 128)	Survivors (*n* = 2592)	*P* value
Age (years) (IQR)	53 (47–60)	67 (58–73)	52 (46–60)	<0.001
Sex				
Male	1530 (56%)	87 (68%)	1443 (56%)	0.006
Female	1190 (44%)	41 (32%)	1149 (44%)	
Body mass index (kg/m^2^)	24 ± 3	24 ± 3	24 ± 3	0.8
<18.5, underweight	86 (3%)	6 (5%)	92 (3%)	0.82
18.5–25, normal	1654 (64%)	82 (64%)	1736 (64%)	
25–30, overweight	817 (30%)	36 (28%)	781 (30%)	
>30, obese	75 (3%)	4 (3%)	71 (3%)	
Smoking status (*n* = 2068)				<0.001
Never smoker	1214 (59%)	42 (42%)	1172 (60%)	
Former smoker	413 (20%)	37 (37%)	376 (19%)	
Current smoker	441 (21%)	21 (21%)	420 (21%)	
Underlying disease				
Cancer history	317 (12%)	39 (31%)	278 (11%)	<0.001
Hypertension	744 (27%)	60 (47%)	684 (26%)	<0.001
Diabetes mellitus	381 (14%)	43 (34%)	338 (13%)	<0.001
Cardiovascular disease	208 (8%)	23 (18%)	185 (7%)	<0.001
Cerebrovascular disease	72 (3%)	10 (8%)	62 (2%)	<0.001
Chronic liver disease	136 (5%)	10 (8%)	126 (5%)	0.14
Chronic renal disease	10 (0.4%)	2 (2%)	8 (0.3%)	0.02
Median follow‐up duration (months) (IQR)	138 (120–153)	103 (67–134)	139 (121–153)	<0.001
Mortality	128 (5%)	128 (5%)	0	
Cancer‐specific mortality	47 (37%)	47 (37%)	0	
Non‐cancer‐specific mortality	81 (63%)	81 (63%)	0	

Abbreviation: IQR, inter‐quartile range.

Significant differences were found between individuals who died and survived in age (median: 67 vs. 52 years; *P* < 0.001), sex (men: 68% vs. 56%; *P* = 0.006), smoking status (never, former and current smokers: 42%, 37% and 21% vs. 60%, 19% and 21%; *P* < 0.001), cancer history (31% vs. 11%; *P* < 0.001), hypertension (47% vs. 26%; *P* < 0.001), DM (34% vs. 13%; *P* < 0.001), cardiovascular disease (18% vs. 7%; *P* < 0.001), cerebrovascular disease (8% vs. 2%; *P* < 0.001), chronic renal disease (2% vs. 0.3%; *P* = 0.02) and follow‐up duration (median: 103 vs. 139 months; *P* < 0.001). However, no significant differences were found for BMI (mean: 24 vs. 24 kg/m^2^; *P* = 0.8) and chronic liver disease (8% vs. 5%; *P* = 0.14).

### Correlations between computed tomography‐derived parameters and body mass index

VFA was negatively correlated with BMI (*r* = −0.62; *P* < 0.001) and VFI (*r* = −0.69; *P* < 0.001). Likewise, SFA was negatively correlated with BMI (*r* = −0.29; *P* < 0.001) and SFI (*r* = −0.62; *P* < 0.001). BMI was positively correlated with VFI (*r* = 0.72; *P* < 0.001) and SFI (*r* = 0.50; *P* < 0.001) (*Table* *2* and *Figure* *S1*).

**Table 2:** Pearson correlation coefficients between visceral and subcutaneous fat attenuation, volume indexes and body mass index

	Pearson correlation coefficient (*r*)	*P* value
Visceral fat attenuation (HU)–body mass index (kg/m^2^)	−0.62	<0.001
Visceral fat attenuation (HU)–visceral fat volume index (cm^3^/m^2^)	−0.69	<0.001
Body mass index (kg/m^2^)–visceral fat volume index (cm^3^/m^2^)	0.72	<0.001
Subcutaneous fat attenuation (HU)–body mass index (kg/m^2^)	−0.29	<0.001
Subcutaneous fat attenuation (HU)–subcutaneous fat index (cm^3^/m^2^)	−0.62	<0.001
Body mass index (kg/m^2^)–subcutaneous fat index (cm^3^/m^2^)	0.50	<0.001

Abbreviation: HU, Hounsfield units.

### Cox regression analyses

**Figure 2:**
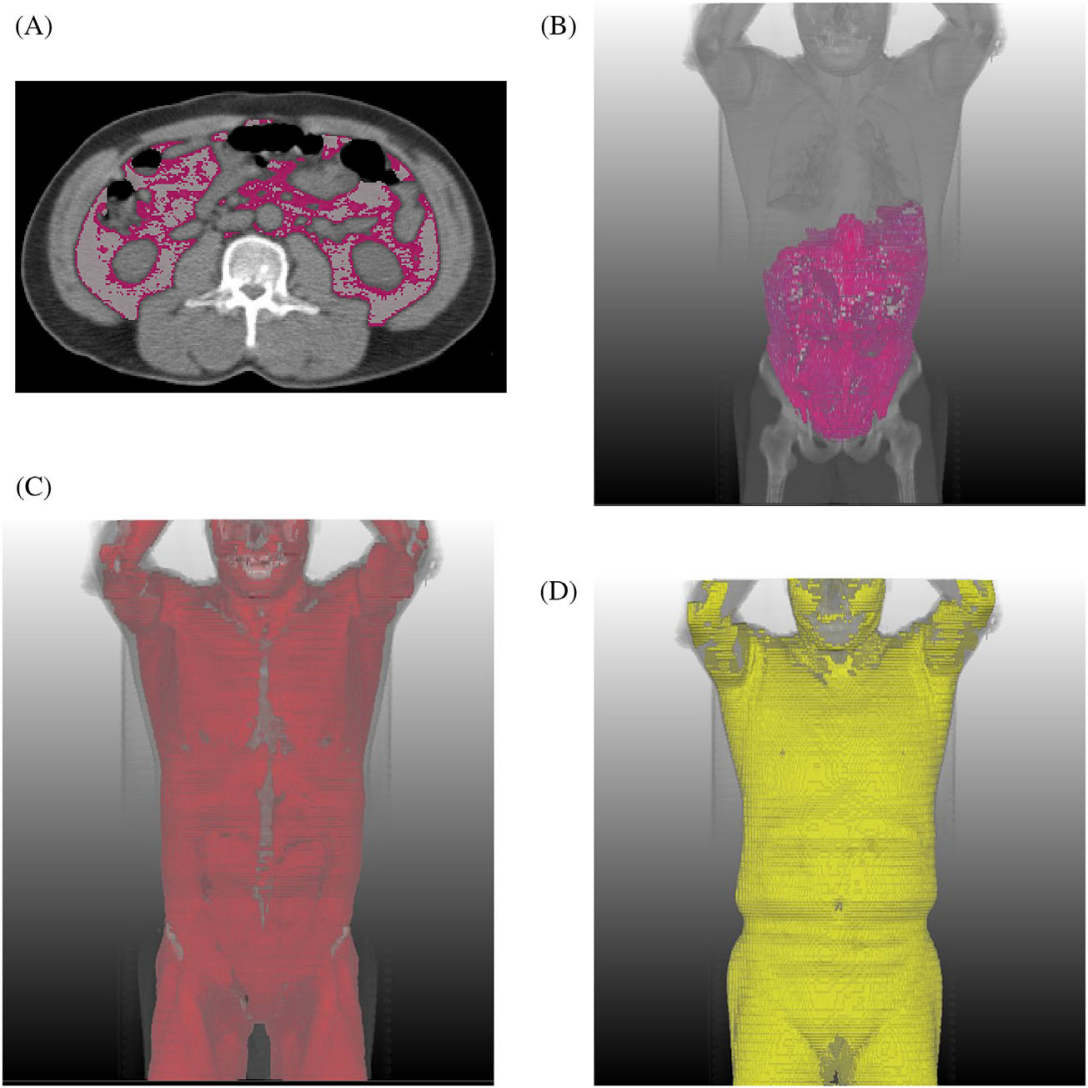
Representative images of high visceral fat attenuation (VFA) predicting long‐term mortality in a 55‐year‐old man with a body mass index of 25.4 kg/m^2^. (A) Segmentation‐overlaid images from body composition analysis of unenhanced axial computed tomography images show the segmentation results of VFA of the highest quartile (pink) and VFA of the lower three quartiles (white). (B–D) Three‐dimensional images from the segmentation showed VFA of the highest quartile (pink in B), VFA of the lower three quartiles (white in B), skeletal muscle (red in C) and subcutaneous fat (yellow in D). This individual's VFA was −85.9 Hounsfield units, which fell into the highest quartile (threshold: −87 Hounsfield units). Ninety‐seven months after the examination, the patient died from pancreatic cancer.

**Figure 3:**
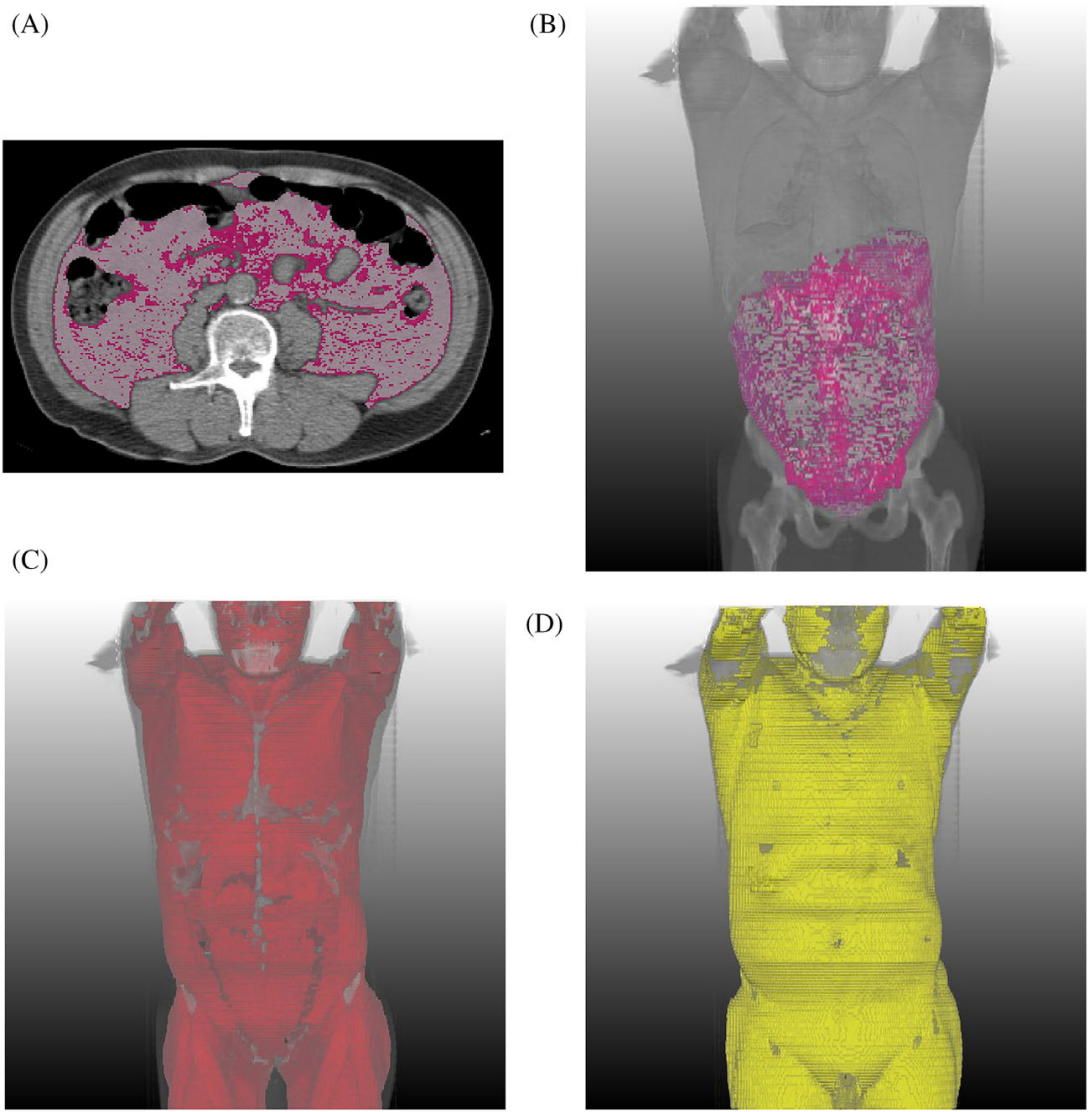
A 67‐year‐old man with a body mass index of 28 kg/m^2^ who underwent a positron‐emission tomography/computed tomography (CT) examination for a health check‐up. (A) Segmentation‐overlaid images from body composition analysis of unenhanced axial CT images show the segmentation results of visceral fat attenuation (VFA) of the highest quartile (pink) and VFA of the lower three quartiles (white). (B–D) Three‐dimensional images from the segmentation showed VFA of the highest quartile (pink in B), VFA of the lower three quartiles (white in B), skeletal muscle (red in C) and subcutaneous fat (yellow in D). This individual's VFA was −99.2 Hounsfield units, falling into the lower three quartiles (threshold: −87 Hounsfield units). He survived as of December 2018 (120 months later).

The results of the Cox regression analyses are described in *Table* *3*. In the univariable Cox regression for OS, significant results were found for VFA (hazard ratio [HR]: 1.03 per HU; 95% confidence interval [CI]: 1.01, 1.06; *P* = 0.003), VFI (HR: 1.002; 95% CI: 1.001, 1.003; *P* = 0.001), SFA (HR: 1.04; 95% CI: 1.02, 1.06; *P* = 0.001), the SFI‐to‐VFI ratio (HR: 0.81; 95% CI: 0.71, 0.93; *P* = 0.003) and sarcopenia (HR: 2.36; 95% CI: 1.6, 3.48; *P* < 0.001). After adjustment for other clinico‐radiological factors in multivariable Cox regression, high VFA (HR: 1.05 per HU; 95% CI: 1.02, 1.08; *P* = 0.001), high VFI (HR: 1.002; 95% CI: 1.00, 1.003; *P* = 0.01) and sarcopenia (HR: 1.73; 95% CI: 1.16, 2.58; *P* = 0.007) were associated with poor OS. After adjusting for clinico‐radiological factors, high VFA was associated with impaired CSS (HR: 1.07 per HU; 95% CI: 1.02, 1.12; *P* = 0.006) and NCS (HR: 1.03 per HU; 95% CI: 1.01, 1.06; *P* = 0.009) (*Figures* *2* and *3*).

**Table 3:** Univariable and multivariable Cox regression analysis for overall survival, cancer‐specific survival and non‐cancer‐specific survival

Variables	Overall survival				Cancer‐specific survival^a^				Non‐cancer‐specific survival^b^			
	Univariable analysis		Multivariable analysis		Univariable analysis		Multivariable analysis		Univariable analysis		Multivariable analysis	
	Hazard ratio	*P* value	Hazard ratio	*P* value	Hazard ratio	*P* value	Hazard ratio	*P* value	Hazard ratio	*P* value	Hazard ratio	*P* value
Age	1.13 (1.11, 1.15)	<0.001	1.11 (1.09, 1.13)	<0.001	1.11 (1.08, 1.14)	<0.001	1.08 (1.05, 1.11)	<0.001	1.15 (1.12, 1.17)	<0.001	1.13 (1.11, 1.16)	<0.001
Sex (reference: male)	0.59 (0.41, 0.86)	0.006			0.49 (0.26, 0.93)	0.03			0.64 (0.41, 1.02)	0.06		
BMI (kg/m^2^)	1.00 (0.94, 1.06)	0.99			1.02 (0.93, 1.12)	0.68			0.99 (0.92, 1.06)	0.74		
BMI (kg/m^2^; reference: 18.5–25, normal)												
<18.5, underweight	1.3 (0.57, 2.98)	0.54			1.23 (0.3, 5.37)	0.74			1.33 (0.48, 3.67)	0.59		
≥25, overweight to obese	0.96 (0.659, 1.41)	0.84			1.12 (0.61, 2.07)	0.72			0.88 (0.54, 1.42)	0.59		
Cancer history	3.72 (2.55, 5.43)	<0.001	2.67 (1.82, 3.93)	<0.001	7.68 (4.30, 13.70)	<0.001	5.88 (3.26, 10.6)	<0.001	2.29 (1.34, 3.91)	0.002		
Hypertension	2.55 (1.80, 3.62)	<0.001			2.04 (1.14, 3.67)	0.02			2.97 (1.92, 4.59)	<0.001		
Diabetes mellitus	3.51 (2.43, 5.07)	<0.001			3.02 (1.61, 5.67)	0.001			3.93 (2.49, 6.19)	<0.001	1.66 (1.04, 2.64)	0.04
Cardiovascular disease	3.04 (1.93, 4.78)	<0.001			3.83 (1.90, 7.73)	<0.001			2.70 (1.49, 4.9)	0.001		
Cerebrovascular disease	3.34 (1.75, 6.38)	<0.001			1.96 (0.48, 8.09)	0.35			4.30 (2.07, 8.94)	<0.001		
Chronic liver disease	1.73 (0.91, 3.29)	0.10			1.41 (0.44, 4.55)	0.56			1.92 (0.88, 4.16)	0.10		
Chronic renal disease	5.47 (1.35, 22.16)	0.02			4.21 (0.03, 29.39)	0.41			8.7 (2.13, 35.55)	0.003	4.79 (1.14, 20.09)	0.03
Sarcopenia (reference: no)^c^	2.36 (1.6, 3.48)	<0.001	1.73 (1.16, 2.58)	0.007	2.61 (1.35, 5.04)	0.004	2.19 (1.13, 4.27)	0.02	2.27 (1.40, 3.68)	0.001	1.61 (0.97, 2.66)	0.07
Fat volume index (cm^3^/m^2^)	1.000 (1.000, 1.001)	0.38			1.000 (0.999, 1.001)	0.49			1.000 (0.999, 1.001)	0.56		
Subcutaneous fat volume index (cm^3^/m^2^)	0.999 (0.998, 1.000)	0.19			0.999 (0.997, 1.001)	0.32			0.999 (0.998, 1.001)	0.34		
Visceral fat volume index (cm^3^/m^2^)	1.002 (1.001, 1.003)	0.001	1.002 (1.00, 1.003)	0.01	1.002 (1.000, 1.003)	0.02	1.003 (1.001, 1.004)	0.006	1.001 (1.000, 1.003)	0.04		
Subcutaneous fat attenuation at the abdominal waist per HU	1.04 (1.02, 1.06)	0.001			1.04 (1.01, 1.07)	0.02			1.04 (1.01, 1.06)	0.01		
Visceral fat attenuation at the abdominal waist per HU	1.03 (1.01, 1.06)	0.003	1.05 (1.02, 1.08)	0.001	1.03 (1.00, 1.07)	0.09	1.07 (1.02, 1.12)	0.006	1.04 (1.01, 1.06)	0.01	1.03 (1.01, 1.06)	0.009
Subcutaneous fat volume index‐to‐visceral fat volume index ratio	0.81 (0.71, 0.93)	0.003			0.78 (0.63, 1.00)	0.05			0.82 (0.7, 0.97)	0.02		

*Note*: Multivariable Cox proportional hazard regression analysis with backward elimination was performed with variables that had *P* values <0.2 in the univariable analysis. The VIFs between variables in multivariable Cox regression analysis were <5.

Abbreviations: BMI, body mass index; HU, Hounsfield units; SMI, skeletal muscle index; VIFs, variance inflation factors.

^a^ Non‐cancer mortality cases were excluded.

^b^ Cancer mortality cases were excluded.

^c^ The cutoff value for lumbar SMI was 55 cm^2^/m^2^ for men and 39 cm^2^/m^2^ for women.

With CT‐derived fat characteristics treated as categorical values, high VFA was also a poor prognostic factor for OS (HR: 1.58 per HU; 95% CI: 1.08, 2.31; *P* = 0.02), CSS (HR: 2.25 per HU; 95% CI: 1.11, 4.59; *P* = 0.03) and NCS (HR: 1.88 per HU; 95% CI: 1.16, 3.03; *P* = 0.01) after adjustment for clinico‐radiological factors (*Table* *S1*). A series of sensitivity analyses, including smoking states by multiple imputations, demonstrated consistent results (*Tables* *S2* and *S3*).

### Visceral fat attenuation and high‐sensitivity C‐reactive protein

A total of 2000 individuals had available hs‐CRP results (median: 0.04 mg/dL; IQR: 0.01–0.14 mg/dL). Individuals with the highest quartile of VFA had significantly higher hs‐CRP levels (median: 0.11 mg/dL; IQR: 0.01–0.2 mg/dL) than those with low VFA (median: 0.03 mg/dL; IQR: 0.01–0.12 mg/dL; *P* < 0.001).

### Sarcopenic obesity

The *C* indexes for OS were 0.52 (95% CI: 0.49, 0.55), 0.58 (95% CI: 0.5, 0.66), 0.55 (95% CI: 0.45, 0.64) and 0.62 (95% CI: 0.51, 0.73) in individuals with SO defined by BMI, VFI, VFA and both VFI and VFA, respectively. The *C* indexes for CSS and NCS were 0.53 (95% CI: 0.49, 0.59) and 0.52 (95% CI: 0.47, 0.56), 0.66 (95% CI: 0.48, 0.84) and 0.55 (95% CI: 0.48, 0.62), 0.53 (95% CI: 0.46, 0.61) and 0.55 (95% CI: 0.41, 0.69), 0.68 (95% CI: 0.53, 0.83) and 0.59 (95% CI: 0.43, 0.75), respectively.

**Figure 4:**
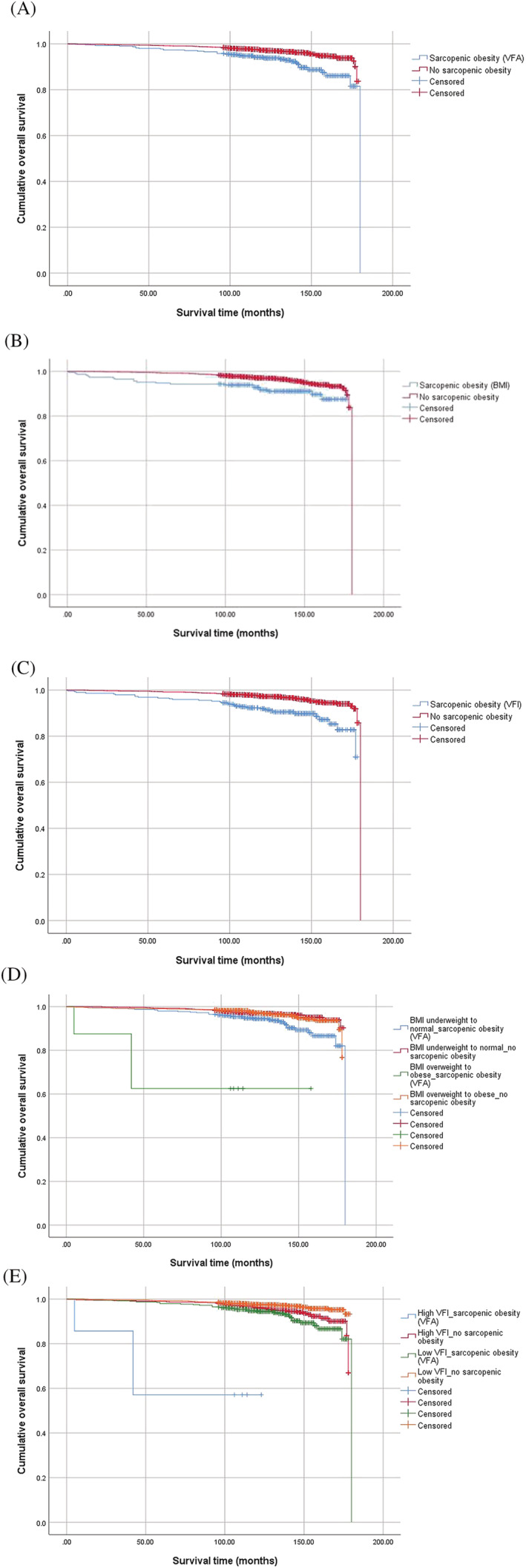
Kaplan–Meier plots for overall survival according to sarcopenic obesity (SO). (A) Individuals with SO defined by visceral fat attenuation (VFA) had poorer outcomes than those without SO (*P* < 0.001). (B) Individuals with SO defined by overweight‐to‐obese body mass index (BMI) had more unfavourable outcomes than those without SO (*P* = 0.001). (C) Individuals with SO defined by the visceral fat volume index (VFI) had poorer outcomes than those without SO (*P* < 0.001). Individuals with SO defined by VFA had poorer long‐term outcomes than those without SO even in the same category of (D) BMI or (E) VFI (all *P* values <0.001).

Individuals with SO defined by VFA (9% vs. 4%; *P* < 0.001), BMI (9% vs. 4%; *P* = 0.001) and VFI (11% vs. 4%; *P* < 0.001) had more unfavourable outcomes than those without SO in OS (*Table* *4* and *Figure* *4*). In the same BMI or VFI category, individuals with SO defined by VFA had poorer OS than those without SO (all *P* values <0.001). The results of the log‐rank test and plots for CSS and NCS are described in *Table* *4* and *Figures* *S2* and *S3*. The results of the log‐rank test for SO defined by visceral fat abnormality and both VFI and BMI are described in *Tables* *S4* and *S5*, respectively.

**Table 4:** Log‐rank test for sarcopenic obesity defined by visceral fat attenuation

Category	Overall mortality		Cancer mortality		Non‐cancer mortality	
	Event	*P* value	Event	*P* value	Event	*P* value
Sarcopenic obesity, defined by VFA						
Sarcopenic obesity	9% (36 of 405)	<0.001	3% (13 of 384)	0.02	6% (24 of 395)	<0.001
No Sarcopenic obesity	4% (92 of 2315)		2% (34 of 2255)		3% (57 of 2278)	
Sarcopenic obesity, defined by BMI						
Sarcopenic obesity	9% (21 of 230)	0.001	5% (11 of 220)	<0.001	5% (10 of 219)	0.2
No Sarcopenic obesity	4% (107 of 2490)		1% (36 of 2419)		3% (71 of 2454)	
Sarcopenic obesity, defined by VFI						
Sarcopenic obesity	11% (32 of 292)	<0.001	5% (15 of 276)	<0.001	6% (17 of 280)	0.002
No Sarcopenic obesity	4% (96 of 2428)		1% (32 of 2363)		3% (64 of 2393)	
In the group with underweight or normal BMI						
Sarcopenia with high VFA^a^	8% (33 of 397)	<0.001	3% (11 of 377)	0.08	6% (23 of 389)	<0.001
No sarcopenia with VFA^a^	4% (55 of 1431)		1% (20 of 1394)		2% (34 of 1408)	
In the group with overweight or obese BMI						
Sarcopenia with high VFA^a^	38% (3 of 8)	<0.001	29% (2 of 7)	<0.001	17% (1 of 6)	0.001
No sarcopenia with VFA^a^	4% (37 of 884)		2% (14 of 861)		3% (23 of 870)	
In the group with high VFI^b^						
Sarcopenia with high VFA^a^	43% (3 of 7)	<0.001	29% (2 of 7)	<0.001	17% (1 of 6)	0.002
No sarcopenia with VFA^a^	6% (39 of 673)		2% (16 of 649)		4% (23 of 656)	
In the group with low VFI^b^						
Sarcopenia with high VFA^a^	8% (33 of 398)	<0.001	3% (11 of 377)	0.02	6% (23 of 389)	<0.001
No sarcopenia with VFA^a^	3% (53 of 1642)		1% (18 of 1606)		2% (34 of 1622)	

*Note*: BMI was categorized as underweight (<18.5 kg/m^2^), normal (18.5–24.9 kg/m^2^), overweight (25–29.9 kg/m^2^) and obese (>30 kg/m^2^).

Abbreviations: BMI, body mass index; VFA, visceral fat attenuation; VFI, visceral fat volume index.

^a^ High VFA defined as the highest quartile.

^b^ High VFI defined as the highest quartile; low VFI defined as the lower three quartiles.

## Discussion

Although fat depots and VFI derived from CT images have been investigated to show their associations with various outcomes, VFA has not been rigorously explored with a volumetric analysis fully capturing the fat distribution in the relevant area. In this study, we investigated the long‐term prognostic implications of VFA derived from CT images using deep learning‐based analysis in a health check‐up population. VFA was negatively correlated with BMI (*r* = −0.62) and VFI (*r* = −0.69). Multivariable Cox analyses suggested that high VFA was associated with poor OS (HR: 1.05 per HU), CSS (HR: 1.07 per HU) and NCS (HR: 1.03 per HU). In addition, individuals with high VFA had significantly higher hs‐CRP levels than those with low VFA (0.11 vs. 0.03 mg/dL; *P* < 0.001), suggesting an underlying mechanism whereby high VFA reflects fat inflammation. Finally, SO defined in terms of VFA stratified individuals' outcomes even in the same category of BMI or VFI (all *P* values <0.05).

Visceral adiposity is an important and complementary barometer of cardiometabolic risk.^7^, ^9^, ^20^, ^21^ Specifically, it has been demonstrated to be associated with cardiovascular events and outcomes, left ventricular remodelling, metabolic diseases including dysglycaemia and insulin resistance.^7^, ^9^, ^21^, ^22^, ^23^, ^24^, ^25^, ^26^, ^27^, ^28^, ^29^ On the contrary, conflicting information persists regarding the implications of VFA for individuals' health. Specifically, some prior studies reported that low VFA was correlated with metabolic syndrome and other adverse cardiovascular risk factors such as impaired fasting glucose and insulin resistance, except DM.^6^, ^8^ However, other studies reported that high VFA was associated with increased all‐cause mortality, cancer mortality and non‐cardiovascular mortality after adjusting for individuals' BMI and VFI.^5^, ^10^ In addition, high VFA was associated with higher levels of coronary and abdominal aortic calcium, which are markers of cardiovascular events and prognosis.^30^ Therefore, our findings that high VFA was associated with lower OS, CSS and NCS are concordant with the latter studies.

Fat attenuation on CT images reflects the output of various underlying cellular and tissue‐level characteristics of adipose tissue. Basically, more lipid‐dense fat tissue and the large size of adipocytes with high lipid droplet content are reflected as low attenuation in CT images,^5^, ^10^, ^31^ which are related to adverse cardiovascular risks.^6^, ^30^ Conversely, abundant vascularization and fibrosis in fat tissue increase attenuation.^6^, ^30^ Interestingly, we found that individuals with high VFA had significantly higher hs‐CRP levels. Because hs‐CRP represents chronic inflammation, which impacts insulin resistance and changes body fat characteristics and volume (i.e., lipid accumulation),^32^, ^33^, ^34^ our findings reflect the chronic inflammation of visceral fat tissue that can be in the midstream of fibrosis,^33^ which can be ultimately correlated to a variety of inflammation‐attributable diseases and mortality.^35^ Indeed, similar results demonstrated that high‐normal levels of hs‐CRP predicted non‐alcoholic fatty liver.^36^ This may be the mechanism underlying the prognostic role of VFA. Nevertheless, it is still unclear whether the association of VFA with hs‐CRP is a simple reflection of unidentified inflammation or the VFA‐originated fibrosis‐related inflammation, for which further research is warranted.

SO is defined as the co‐existence of sarcopenia and obesity, which synergistically worsen one another.^37^, ^38^ SO is an emerging public health problem, causing negative consequences including disability, comorbidities such as DM and increased mortality.^37^, ^38^, ^39^ Although obesity in defining SO is basically based on the BMI, BMI only reflects the body weight, rather than adiposity, and cannot distinguish body fat from muscle or bone.^4^, ^37^, ^38^ In our study, we applied VFA to define obesity in SO, and OS, CSS and NCS were significantly different according to the presence of SO, even for individuals in the same BMI or VFI category. This result suggests the prognostic usefulness of VFA in defining obesity in SO.

Several limitations of this study should be mentioned. First, this study was retrospectively performed at a single centre with a single‐ethnicity study population. Second, we could not perform sex‐specific analyses because of the sparse number of events. Third, although various body composition parameters, including fat volume and quality, are associated with comorbidities such as metabolic syndrome,^6^, ^8^ we only set survival as the outcome. Fourth, we investigated the initial CT images for individuals, not serial changes in body composition with their follow‐up images. However, because serial changes in adiposity are associated with serum lipoprotein levels and cardiovascular risk,^40^ further research is warranted to confirm the impact of changes in body composition on long‐term outcomes. Finally, because the segmentation results of other deep learning‐based body composition analysis software could differ from our results, our results cannot represent all other body composition analysis software.

In conclusion, high VFA was associated with long‐term mortality and low‐grade inflammation. VFA can further stratify the current SO by BMI or VFI, and SO defined by VFA can identify individuals who are most vulnerable to long‐term mortality due to visceral inflammatory obesity, which has not been possible to date using BMI and visceral fat measurements.

## Funding

This work was supported by the Korea Medical Device Development Fund grant funded by the Korean government (the Ministry of Science and ICT, the Ministry of Trade, Industry and Energy, the Ministry of Health & Welfare, Republic of Korea, and the Ministry of Food and Drug Safety) (Project Number 202011A03). The funding source had no role in the study design, data collection and analysis, decision to publish or preparation of this study.

## Conflict of interest statement

Soon Ho Yoon is a chief medical officer for Medical IP and holds a stock of the firm. Jong Hyuk Lee, Seung Ho Choi, Keum Ji Jung and Jin Mo Goo declare that they have no conflicts of interest.

## Supporting information


**Data S1.** Supplementary References

**Figure S1.** Plots of Pearson correlation coefficients between visceral or subcutaneous fat attenuation, fat volume index, and body mass index. (A) Visceral fat attenuation and body mass index, (B) visceral fat attenuation and visceral fat volume index, (C) visceral fat volume index and body mass index, (D) subcutaneous fat attenuation and body mass index, (B) subcutaneous fat attenuation and visceral fat volume index, (C) subcutaneous fat volume index and body mass index
**Figure S2.** Kaplan–Meier plots for cancer‐specific survival according to sarcopenic obesity (SO). (A) Individuals with SO defined by visceral fat attenuation (VFA) had poorer outcomes than those without SO (*P* = 0.02); (B) individuals with SO defined by overweight‐to‐obese body mass index (BMI) had more unfavorable outcomes than those without SO (*P* < 0.001); (C) individuals with SO defined by the visceral fat volume index (VFI) had poorer outcomes than those without SO (*P* < 0.001); individuals with SO defined by VFA had poorer long‐term outcomes than those without SO even in the same category of BMI (D) or VFI (E) (all *P*‐values <0.05) except in individuals with underweight or normal BMI (*P* = 0.08)
**Figure S3.** Kaplan–Meier plots for non‐cancer‐specific survival according to sarcopenic obesity (SO). (A) Individuals with SO defined by visceral fat attenuation (VFA) had poorer outcomes than those without SO (*P* < 0.001); (B) No significant differences in outcomes were observed between individuals with SO defined by an overweight to obese body mass index (BMI) and those without SO (*P* = 0.2); (C) Individuals with SO defined by the visceral fat volume index (VFI) had poorer outcomes than those without SO (*P* = 0.002); individuals with SO defined by VFA had poorer long‐term outcomes than those without SO, even in the same category of BMI (D) or VFI (E) (all *P*‐values <0.05)

**Table S1.** Univariable and multivariable Cox regression analysis for overall survival, cancer‐specific survival, and non‐cancer‐specific survival, in which CT‐derived parameters were treated as categorical variables
**Table S2.** Univariable and multivariable Cox regression analysis for overall survival, cancer‐specific survival, and non‐cancer‐specific survival with multiple imputation of smoking status.
**Table S3.** Univariable and multivariable Cox regression analysis for overall survival, cancer‐specific survival, and non‐cancer‐specific survival with multiple imputation of smoking status, in which CT‐derived parameters were treated as categorical variables
**Table S4.** Log‐rank test for sarcopenic obesity defined by visceral fat abnormality
**Table S5.** Log‐rank test for sarcopenic obesity defined by the visceral fat volume index within the same body mass index category
