# Supplementary material for: High visceral fat attenuation and long‐term mortality in a health check‐up population
Source: J Cachexia Sarcopenia Muscle. 2023 Apr 5;14(3):1495–507. doi: 10.1002/jcsm.13226 (PMC10235877; doi:10.1002/jcsm.13226)
Supplement: Supplementary file 1 — Data S1. Supplementary References [file JCSM-14-1495-s002.docx]

**Supplementary References**

Article title: High Visceral Fat Attenuation and Long-term Mortality in a Health Check-up Population

Journal name: Journal of Cachexia, Sarcopenia and Muscle

Author names: Jong Hyuk Lee, MD, PhD Seung Ho Choi, MD, PhD Keum Ji Jung, PhD Jin Mo Goo, MD, PhD^6^ and Soon Ho Yoon, MD, PhD

Address correspondence to: Soon Ho Yoon, MD, PhD.

Department of Radiology, Seoul National University Hospital, Seoul National College of Medicine, 101 Daehak-ro, Jongno-gu, Seoul 03080, Korea; Telephone: 82-2-2072-2254; Fax: 82-2-743-6385; E-mail: [yshoka@gmail.com](mailto:yshoka@gmail.com)

S1. Choi H, Park YS, Na KJ, et al. Association of Adipopenia at Preoperative PET/CT with Mortality in Stage I Non-Small Cell Lung Cancer. Radiology. 2021; 301(3):645-653.

S2. Lee JH, Hyung S, Lee J, Choi S. Visceral adiposity and systemic inflammation in the obesity paradox in patients with unresectable or metastatic melanoma undergoing immune checkpoint inhibitor therapy: a retrospective cohort study. J Immunother Cancer. 2022;10(8):e005226. doi: 10.1136/jitc-2022-005226.

S3. Kim SI, Yoon SH, Kim TM, Cho JY, Chung HH, Song YS. Prognostic implications of body composition change during primary treatment in patients with ovarian cancer: A retrospective study using an artificial intelligence-based volumetric technique. Gynecol Oncol. 2021;162(1):72-79.

S4. Uno H, Cai T, Pencina MJ, D’Agostino RB, Wei LJ. On the C-statistics for evaluating overall adequacy of risk prediction procedures with censored survival data. Stat Med. 2011;30(10):1105-1117.

S5. Uno H, Cai T. Tian L, Wei LJ. Evaluating Prediction Rules for t-Year Survivors with Censored Regression Models. J Am Stat Assoc. 2007;102: 527–537.
