# Supplementary material for: High visceral fat attenuation and long‐term mortality in a health check‐up population
Source: J Cachexia Sarcopenia Muscle. 2023 Apr 5;14(3):1495–507. doi: 10.1002/jcsm.13226 (PMC10235877; doi:10.1002/jcsm.13226)

**Supporting Figures**

Article title: High Visceral Fat Attenuation and Long-term Mortality in a Health Check-up Population

Journal name: Journal of Cachexia, Sarcopenia and Muscle

Author names: Jong Hyuk Lee, MD, PhD Seung Ho Choi, MD, PhD Keum Ji Jung, PhD Jin Mo Goo, MD, PhD^6^ and Soon Ho Yoon, MD, PhD

Address correspondence to: Soon Ho Yoon, MD, PhD.

Department of Radiology, Seoul National University Hospital, Seoul National College of Medicine, 101 Daehak-ro, Jongno-gu, Seoul 03080, Korea; Telephone: 82-2-2072-2254; Fax: 82-2-743-6385; E-mail: yshoka@gmail.com

**Figure S1** Plots of Pearson correlation coefficients between visceral or subcutaneous fat attenuation, fat volume index, and body mass index. (A) Visceral fat attenuation and body mass index, (B) visceral fat attenuation and visceral fat volume index, (C) visceral fat volume index and body mass index, (D) subcutaneous fat attenuation and body mass index, (B) subcutaneous fat attenuation and visceral fat volume index, (C) subcutaneous fat volume index and body mass index


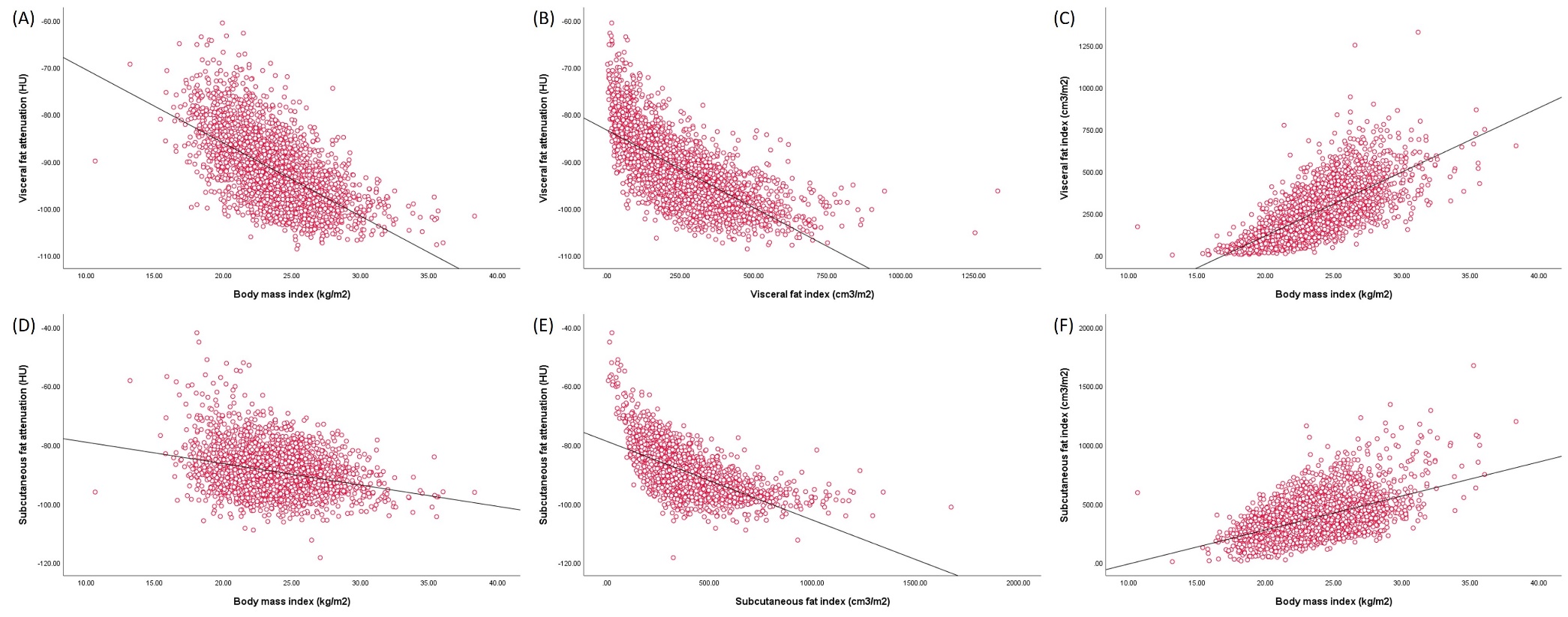


**Figure S2** Kaplan-Meier plots for cancer-specific survival according to sarcopenic obesity (SO). (A) Individuals with SO defined by visceral fat attenuation (VFA) had poorer outcomes than those without SO (*P* =0.02); (B) individuals with SO defined by overweight-to-obese body mass index (BMI) had more unfavorable outcomes than those without SO (*P* <0.001); (C) individuals with SO defined by the visceral fat volume index (VFI) had poorer outcomes than those without SO (*P* <0.001); individuals with SO defined by VFA had poorer long-term outcomes than those without SO even in the same category of BMI (D) or VFI (E) (all *P*-values <0.05) except in individuals with underweight or normal BMI (*P* =0.08)


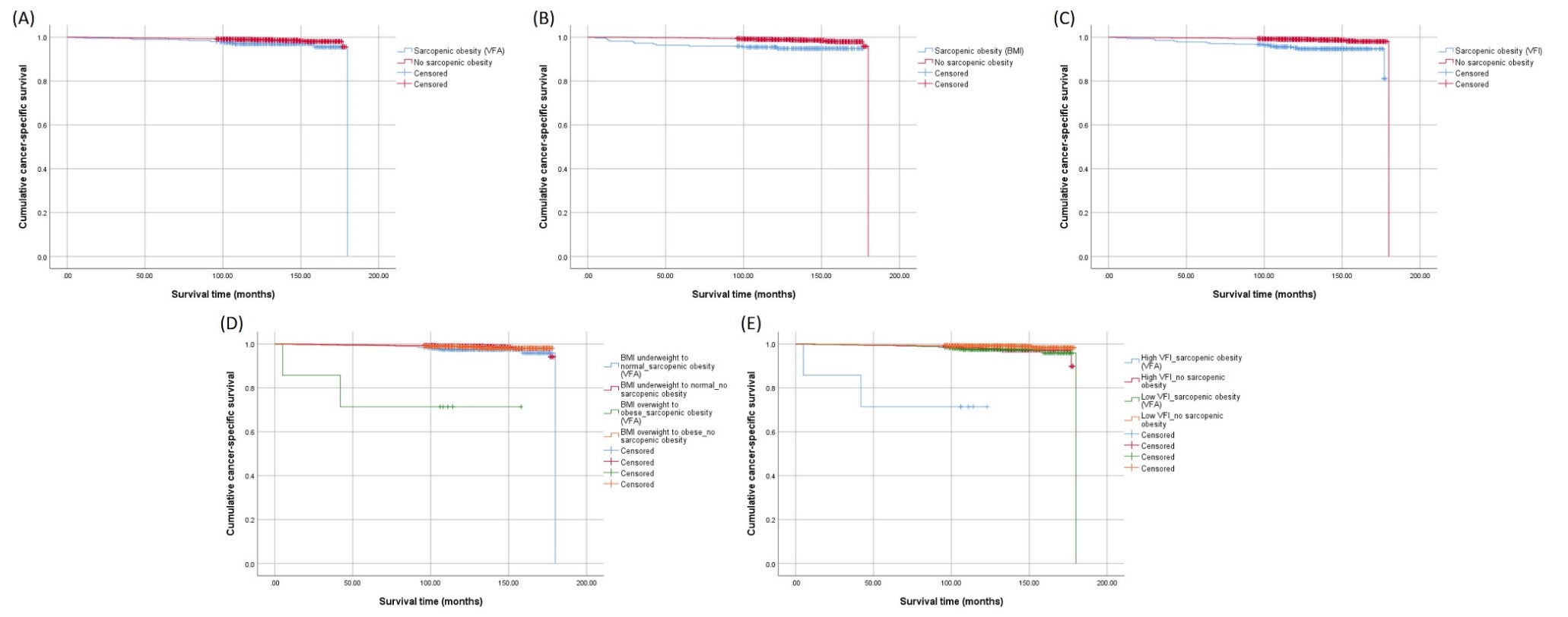


**Figure S3** Kaplan-Meier plots for non-cancer-specific survival according to sarcopenic obesity (SO). (A) Individuals with SO defined by visceral fat attenuation (VFA) had poorer outcomes than those without SO (*P* <0.001); (B) No significant differences in outcomes were observed between individuals with SO defined by an overweight to obese body mass index (BMI) and those without SO (*P* =0.2); (C) Individuals with SO defined by the visceral fat volume index (VFI) had poorer outcomes than those without SO (*P* =0.002); individuals with SO defined by VFA had poorer long-term outcomes than those without SO, even in the same category of BMI (D) or VFI (E) (all *P*-values <0.05)


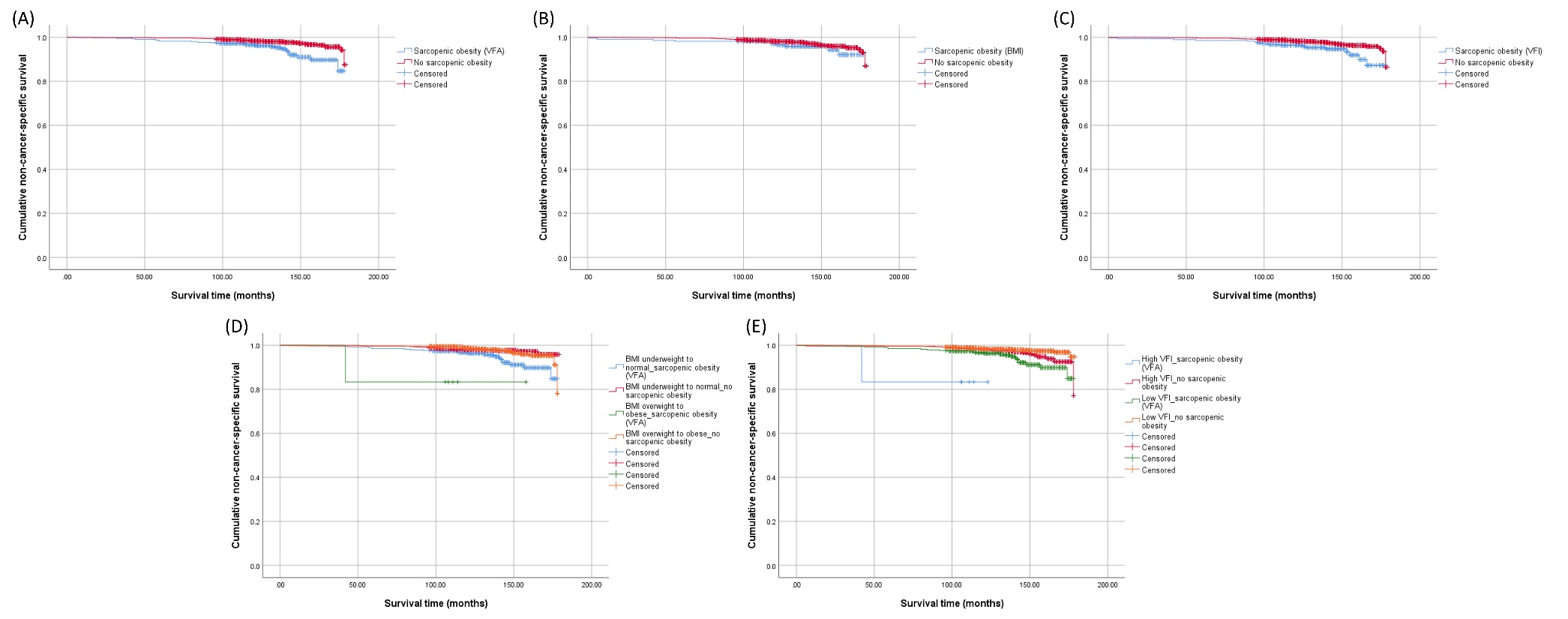

Supplement: Supplementary file 2 — Figure S1. Plots of Pearson correlation coefficients between visceral or subcutaneous fat attenuation, fat volume index, and body mass index. (A) Visceral fat attenuation and body mass index, (B) visceral fat attenuation and visceral fat volume index, (C) visceral fat volume index and body mass index, (D) subcutaneous fat attenuation and body mass index, (B) subcutaneous fat attenuation and visceral fat volume index, (C) subcutaneous fat volume index and body mass index Figure S2. Kaplan–Meier plots for cancer‐specific survival according to sarcopenic obesity (SO). (A) Individuals with SO defined by visceral fat attenuation (VFA) had poorer outcomes than those without SO (P = 0.02); (B) individuals with SO defined by overweight‐to‐obese body mass index (BMI) had more unfavorable outcomes than those without SO (P < 0.001); (C) individuals with SO defined by the visceral fat volume index (VFI) had poorer outcomes than those without SO (P < 0.001); individuals with SO defined by VFA had poorer long‐term outcomes than those without SO even in the same category of BMI (D) or VFI (E) (all P‐values <0.05) except in individuals with underweight or normal BMI (P = 0.08) Figure S3. Kaplan–Meier plots for non‐cancer‐specific survival according to sarcopenic obesity (SO). (A) Individuals with SO defined by visceral fat attenuation (VFA) had poorer outcomes than those without SO (P < 0.001); (B) No significant differences in outcomes were observed between individuals with SO defined by an overweight to obese body mass index (BMI) and those without SO (P = 0.2); (C) Individuals with SO defined by the visceral fat volume index (VFI) had poorer outcomes than those without SO (P = 0.002); individuals with SO defined by VFA had poorer long‐term outcomes than those without SO, even in the same category of BMI (D) or VFI (E) (all P‐values <0.05) [file JCSM-14-1495-s001.docx]
