# Supplementary material for: High visceral fat attenuation and long‐term mortality in a health check‐up population
Source: J Cachexia Sarcopenia Muscle. 2023 Apr 5;14(3):1495–507. doi: 10.1002/jcsm.13226 (PMC10235877; doi:10.1002/jcsm.13226)
Supplement: Supplementary file 3 — Table S1. Univariable and multivariable Cox regression analysis for overall survival, cancer‐specific survival, and non‐cancer‐specific survival, in which CT‐derived parameters were treated as categorical variables Table S2. Univariable and multivariable Cox regression analysis for overall survival, cancer‐specific survival, and non‐cancer‐specific survival with multiple imputation of smoking status. Table S3. Univariable and multivariable Cox regression analysis for overall survival, cancer‐specific survival, and non‐cancer‐specific survival with multiple imputation of smoking status, in which CT‐derived parameters were treated as categorical variables Table S4. Log‐rank test for sarcopenic obesity defined by visceral fat abnormality Table S5. Log‐rank test for sarcopenic obesity defined by the visceral fat volume index within the same body mass index category [file JCSM-14-1495-s003.docx]

Supporting Tables

Article title: High Visceral Fat Attenuation and Long-term Mortality in a Health Check-up Population

Journal name: Journal of Cachexia, Sarcopenia and Muscle

Author names: Jong Hyuk Lee, MD, PhD Seung Ho Choi, MD, PhD Keum Ji Jung, PhD Jin Mo Goo, MD, PhD^6^ and Soon Ho Yoon, MD, PhD

Address correspondence to: Soon Ho Yoon, MD, PhD.

Department of Radiology, Seoul National University Hospital, Seoul National College of Medicine, 101 Daehak-ro, Jongno-gu, Seoul 03080, Korea; Telephone: 82-2-2072-2254; Fax: 82-2-743-6385; E-mail: yshoka@gmail.com

Table S1. Univariable and multivariable Cox regression analysis for overall survival, cancer-specific survival, and non-cancer-specific survival, in which CT-derived parameters were treated as categorical variables

|  | Overall survival | | | | Cancer-specific survival† | | | | Non-cancer-specific survival‡ | | | |
| --- | --- | --- | --- | --- | --- | --- | --- | --- | --- | --- | --- | --- |
|  | Univariable analysis | | Multivariable analysis | | Univariable analysis | | Multivariable analysis | | Univariable analysis | | Multivariable analysis | |
| Variables | Hazard ratio | *P* value | Hazard ratio | *P* value | Hazard ratio | *P* value | Hazard ratio | *P* value | Hazard ratio | *P* value | Hazard ratio | *P* value |
| Age | 1.13 (1.11, 1.15) | <0.001 | 1.11 (1.09, 1.13) | <0.001 | 1.11 (1.08, 1.14) | <0.001 | 1.09 (1.06, 1.12) | <0.001 | 1.15 (1.12, 1.17) | <0.001 | 1.13 (1.11, 1.16) | <0.001 |
| Sex (reference: male) | 0.59 (0.41, 0.86) | 0.006 |  |  | 0.49 (0.26, 0.93) | 0.03 |  |  | 0.64 (0.41, 1.02) | 0.06 |  |  |
| BMI (kg/m^2^) | 1.00 (0.94, 1.06) | 0.99 |  |  | 1.02 (0.93, 1.12) | 0.68 |  |  | 0.99 (0.92, 1.06) | 0.74 |  |  |
| BMI (kg/m^2^; reference: 18.5-25, normal) |  |  |  |  |  |  |  |  |  |  |  |  |
| <18.5, underweight | 1.3 (0.57, 2.98) | 0.54 |  |  | 1.23 (0.3, 5.37) | 0.74 |  |  | 1.33 (0.48, 3.67) | 0.59 |  |  |
| ≥25, overweight to obese | 0.96 (0.659, 1.41) | 0.84 |  |  | 1.12 (0.61, 2.07) | 0.72 |  |  | 0.88 (0.54, 1.42) | 0.59 |  |  |
| Cancer history | 3.72 (2.55, 5.43) | <0.001 | 2.65 (1.8, 3.89) | <0.001 | 7.68 (4.30, 13.7) | <0.001 | 6.48 (3.58, 11.70) | <0.001 | 2.29 (1.34 3.91) | 0.002 |  |  |
| Hypertension | 2.55 (1.80, 3.62) | <0.001 |  |  | 2.04 (1.14, 3.67) | 0.02 |  |  | 2.97 (1.92, 4.59) | <0.001 |  |  |
| Diabetes mellitus | 3.51 (2.43, 5.07) | <0.001 | 1.50 (1.02, 2.20) | 0.04 | 3.02 (1.61, 5.67) | 0.001 |  |  | 3.93 (2.49, 6.19) | <0.001 | 1.66 (1.04, 2.66) | 0.03 |
| Cardiovascular disease | 3.04 (1.93, 4.78) | <0.001 |  |  | 3.83 (1.9, 7.73) | <0.001 |  |  | 2.7 (1.49, 4.90) | 0.001 |  |  |
| Cerebrovascular disease | 3.34 (1.75, 6.38) | <0.001 |  |  | 1.96 (0.48, 8.09) | 0.35 |  |  | 4.3 (2.07, 8.94) | <0.001 |  |  |
| Chronic liver disease | 1.73 (0.91, 3.29) | 0.10 |  |  | 1.41 (0.44, 4.55) | 0.56 |  |  | 1.92 (0.88, 4.16) | 0.10 |  |  |
| Chronic renal disease | 5.47 (1.35, 22.16) | 0.02 |  |  | 4.21 (0.03, 29.39) | 0.41 |  |  | 8.7 (2.13, 35.55) | 0.003 | 4.53 (1.08, 19.02) | 0.04 |
| Sarcopenia (reference: no)* | 2.36 (1.60, 3.48) | <0.001 | 1.67 (1.12, 2.48) | 0.01 | 2.61 (1.35, 5.04) | 0.004 | 2.16 (1.11, 4.21) | 0.02 | 2.27 (1.40, 3.68) | 0.001 | 1.63 (0.99, 2.69) | 0.06 |
| Fat volume index (reference: lower three quartiles) | 1.00 (0.67, 1.49) | >0.999 |  |  | 1.18 (0.62, 2.25) | 0.61 |  |  | 0.91 (0.54, 1.51) | 0.70 |  |  |
| Subcutaneous fat volume index (reference: lower three quartiles) | 0.79 (0.51, 2.21) | 0.27 |  |  | 0.84 (0.42, 1.70) | 0.63 |  |  | 0.75 (0.43, 1.30) | 0.30 |  |  |
| Visceral fat volume index (reference: lower three quartiles) | 1.53 (1.05, 2.21) | 0.03 |  |  | 1.98 (1.09, 3.58) | 0.02 | 2.44 (1.22, 4.86) | 0.01 | 1.29 (0.80, 2.07) | 0.30 |  |  |
| Subcutaneous fat attenuation (reference: lower three quartiles) | 1.74 (1.19, 2.53) | 0.004 |  |  | 1.67 (0.90, 3.10) | 0.11 |  |  | 1.78 (1.10, 2.86) | 0.02 |  |  |
| Visceral fat attenuation (reference: lower three quartiles) | 1.55 (1.07, 2.26) | 0.02 | 1.58 (1.08, 2.31) | 0.02 | 1.55 (0.84, 2.87) | 0.16 | 2.25 (1.11, 4.59) | 0.03 | 1.65 (1.03, 2.63) | 0.04 | 1.88 (1.16, 3.03) | 0.01 |
| Subcutaneous fat volume index to visceral fat volume index ratio (reference: lower three quartiles) | 0.56 (0.34, 0.90) | 0.02 |  |  | 0.45 (0.19, 1.06) | 0.07 |  |  | 0.62 (0.35, 1.11) | 0.11 |  |  |

BMI: body mass index

Multivariable Cox proportional hazard regression analysis with backward elimination was performed with variables that had *P*-values <0.2 in the univariable analysis.

The variance inflation factors (VIF) between variables in multivariable Cox regression analysis were <5

* The cutoff value for lumbar SMI was 55 cm^2^/m^2^ for men and 39 cm^2^/m^2^ for women

† Non-cancer mortality cases were excluded.

‡ Cancer mortality cases were excluded.

Table S2. Univariable and multivariable Cox regression analysis for overall survival, cancer-specific survival, and non-cancer-specific survival with multiple imputation of smoking status.

|  | Overall survival | | | | Cancer-specific survival† | | | | Non-cancer-specific survival‡ | | | |
| --- | --- | --- | --- | --- | --- | --- | --- | --- | --- | --- | --- | --- |
|  | Univariable analysis | | Multivariable analysis | | Univariable analysis | | Multivariable analysis | | Univariable analysis | | Multivariable analysis | |
| Variables | Hazard ratio | *P* value | Hazard ratio | *P* value | Hazard ratio | *P* value | Hazard ratio | *P* value | Hazard ratio | *P* value | Hazard ratio | *P* value |
| Age | 1.13 (1.11, 1.15) | <0.001 | 1.11 (1.09, 1.14) | <0.001 | 1.11 (1.08, 1.14) | <0.001 | 1.09 (1.06, 1.13) | <0.001 | 1.15 (1.12, 1.17) | <0.001 | 1.14 (1.11, 1.16) | <0.001 |
| Sex (reference: male) | 0.59 (0.41, 0.86) | 0.006 |  |  | 0.49 (0.26, 0.93) | 0.03 |  |  | 0.64 (0.41, 1.02) | 0.06 |  |  |
| BMI (kg/m^2^) | 1.00 (0.94, 1.06) | 0.99 |  |  | 1.02 (0.93, 1.12) | 0.68 |  |  | 0.99 (0.92, 1.06) | 0.74 |  |  |
| BMI (kg/m^2^; reference: 18.5-25, normal) |  |  |  |  |  |  |  |  |  |  |  |  |
| <18.5, underweight | 1.3 (0.57, 2.98) | 0.54 |  |  | 1.23 (0.3, 5.37) | 0.74 |  |  | 1.33 (0.48, 3.67) | 0.59 |  |  |
| >25, overweight to obese | 0.96 (0.659, 1.41) | 0.84 |  |  | 1.12 (0.61, 2.07) | 0.72 |  |  | 0.88 (0.54, 1.42) | 0.59 |  |  |
| Smoking status (reference: never smoker) § |  |  |  |  |  |  |  |  |  |  |  |  |
| Former smoker | 2.47 (1.61, 3.79) | <0.001 | 1.12 (0.72, 1.76) | 0.61 | 2.79 (1.26, 6.19) | 0.01 | 1.20 (0.52, 2.78) | 0.67 | 2.38 (1.44, 3.92) | 0.001 |  |  |
| Current smoker | 1.32 (0.82, 2.15) | 0.26 | 1.88 (1.09, 3.26) | 0.03 | 2.23 (1.03, 4.83) | 0.04 | 2.78 (1.22, 6.35) | 0.02 | 0.93 (0.46, 1.90) | 0.85 |  |  |
| Cancer history | 3.72 (2.55, 5.43) | <0.001 | 2.71 (1.84, 4.00) | <0.001 | 7.68 (4.3, 13.7) | <0.001 | 6.15 (3.4, 11.12) | <0.001 | 2.29 (1.34 3.91) | 0.002 |  |  |
| Hypertension | 2.55 (1.80, 3.62) | <0.001 |  |  | 2.04 (1.14, 3.67) | 0.02 |  |  | 2.97 (1.92, 4.59) | <0.001 |  |  |
| Diabetes mellitus | 3.51 (2.43, 5.07) | <0.001 |  |  | 3.02 (1.61, 5.67) | 0.001 |  |  | 3.93 (2.49, 6.19) | <0.001 | 1.68 (1.05, 2.68) | 0.03 |
| Cardiovascular disease | 3.04 (1.93, 4.78) | <0.001 |  |  | 3.83 (1.9, 7.73) | <0.001 |  |  | 2.70 (1.49, 4.9) | 0.001 |  |  |
| Cerebrovascular disease | 3.34 (1.75, 6.38) | <0.001 |  |  | 1.96 (0.48, 8.09) | 0.35 |  |  | 4.30 (2.07, 8.94) | <0.001 |  |  |
| Chronic liver disease | 1.73 (0.91, 3.29) | 0.10 |  |  | 1.41 (0.44, 4.55) | 0.56 |  |  | 1.92 (0.88, 4.16) | 0.10 |  |  |
| Chronic renal disease | 5.47 (1.35, 22.16) | 0.02 |  |  | 4.21 (0.03, 29.39) | 0.41 |  |  | 8.70 (2.13, 35.55) | 0.003 |  |  |
| Sarcopenia (reference: no)* | 2.36 (1.6, 3.48) | <0.001 | 1.62 (1.08, 2.43) | 0.02 | 2.61 (1.35, 5.04) | 0.004 | 1.95 (0.99, 3.85) | 0.06 | 2.27 (1.4, 3.68) | 0.001 |  |  |
| Fat volume index (cm^3^/m^2^) | 1.000 (1.000, 1.001) | 0.38 |  |  | 1.000 (0.999, 1.001) | 0.49 |  |  | 1.000 (0.999, 1.001) | 0.56 |  |  |
| Subcutaneous fat volume index (cm^3^/m^2^) | 0.999 (0.998, 1.000) | 0.19 |  |  | 0.999 (0.997, 1.001) | 0.32 |  |  | 0.999 (0.998, 1.001) | 0.34 |  |  |
| Visceral fat volume index (cm^3^/m^2^) | 1.002 (1.001, 1.003) | 0.001 | 1.001 (1.000, 1.003) | 0.04 | 1.002 (1.000, 1.003 | 0.02 | 1.002 (1.000, 1.004) | 0.03 | 1.001 (1.000, 1.003) | 0.04 |  |  |
| Subcutaneous fat attenuation at the abdominal waist per a HU | 1.04 (1.02, 1.06) | 0.001 |  |  | 1.04 (1.01, 1.07) | 0.02 |  |  | 1.04 (1.01, 1.06) | 0.01 |  |  |
| Visceral fat attenuation at the abdominal waist per a HU | 1.03 (1.01, 1.06) | 0.003 | 1.05 (1.02, 1.08) | 0.002 | 1.03 (1.00, 1.07) | 0.09 | 1.06 (1.01, 1.11) | 0.02 | 1.04 (1.01, 1.06) | 0.01 | 1.04 (1.01, 1.06) | 0.005 |
| Subcutaneous fat volume index to visceral fat volume index ratio | 0.81 (0.71, 0.93) | 0.003 |  |  | 0.78 (0.63, 1.00) | 0.05 |  |  | 0.82 (0.70, 0.97) | 0.02 |  |  |

BMI: body mass index

Multivariable Cox proportional hazard regression analysis with backward elimination was performed with variables that had *P*-values <0.2 in the univariable analysis.

The variance inflation factors (VIF) between variables in multivariable Cox regression analysis were <5

* The cutoff value for lumbar SMI was 55 cm^2^/m^2^ for men and 39 cm^2^/m^2^ for women

† Non-cancer mortality cases were excluded.

‡ Cancer mortality cases were excluded.

§ Multiple imputation was performed for smoking status.

Table S3. Univariable and multivariable Cox regression analysis for overall survival, cancer-specific survival, and non-cancer-specific survival with multiple imputation of smoking status, in which CT-derived parameters were treated as categorical variables

|  | Overall survival | | | | Cancer-specific survival† | | | | Non-cancer-specific survival‡ | | | |
| --- | --- | --- | --- | --- | --- | --- | --- | --- | --- | --- | --- | --- |
|  | Univariable analysis | | Multivariable analysis | | Univariable analysis | | Multivariable analysis | | Univariable analysis | | Multivariable analysis | |
| Variables | Hazard ratio | *P* value | Hazard ratio | *P* value | Hazard ratio | *P* value | Hazard ratio | *P* value | Hazard ratio | *P* value | Hazard ratio | *P* value |
| Age | 1.13 (1.11, 1.15) | <0.001 | 1.12 (1.10, 1.14) | <0.001 | 1.11 (1.08, 1.14) | <0.001 | 1.104 (1.068, 1.14) | <0.001 | 1.15 (1.12, 1.17) | <0.001 | 1.14 (1.11, 1.16) | <0.001 |
| Sex (reference: male) | 0.59 (0.41, 0.86) | 0.006 |  |  | 0.49 (0.26, 0.93) | 0.03 |  |  | 0.64 (0.41, 1.02) | 0.06 |  |  |
| BMI (kg/m^2^) | 1.00 (0.94, 1.06) | 0.99 |  |  | 1.02 (0.93, 1.12) | 0.68 |  |  | 0.99 (0.92, 1.06) | 0.74 |  |  |
| BMI (kg/m^2^; reference: 18.5-25, normal) |  |  |  |  |  |  |  |  |  |  |  |  |
| <18.5, underweight | 1.3 (0.57, 2.98) | 0.54 |  |  | 1.23 (0.3, 5.37) | 0.74 |  |  | 1.33 (0.48, 3.67) | 0.59 |  |  |
| ≥25, overweight to obese | 0.96 | 0.84 |  |  | 1.12 (0.61, 2.07) | 0.72 |  |  | 0.88 (0.54, 1.42) | 0.59 |  |  |
| Smoking status (reference: never smoker) § |  |  |  |  |  |  |  |  |  |  |  |  |
| Former smoker | 2.47 (1.61, 3.79) | <0.001 | 1.02 (0.61, 1.69) | 0.95 | 2.79 (1.26, 6.19) | 0.01 | 1.39 (0.62, 3.13) | 0.42 | 2.38 (1.44, 3.92) | 0.001 |  |  |
| Current smoker | 1.32 (0.82, 2.15) | 0.26 | 1.92 (1.11, 3.32) | 0.02 | 2.23 (1.03, 4.83) | 0.04 | 3.37 (1.52, 7.49) | 0.003 | 0.93 (0.46, 1.9) | 0.85 |  |  |
| Cancer history | 3.72 (2.55, 5.43) | <0.001 | 2.68 (1.82, 3.94) | <0.001 | 7.68 (4.30, 13.70) | <0.001 | 6.71 (3.70, 12.16) | <0.001 | 2.29 (1.34 3.91) | 0.002 |  |  |
| Hypertension | 2.55 (1.8, 3.62) | <0.001 |  |  | 2.04 (1.14, 3.67) | 0.02 |  |  | 2.97 (1.92, 4.59) | <0.001 |  |  |
| Diabetes mellitus | 3.51 (2.43, 5.07) | <0.001 | 1.46 (0.94, 2.27) | 0.09 | 3.02 (1.61, 5.67) | 0.001 |  |  | 3.93 (2.49, 6.19) | <0.001 | 1.69 (1.06, 2.70) | 0.03 |
| Cardiovascular disease | 3.04 (1.93, 4.78) | <0.001 |  |  | 3.83 (1.9, 7.73) | <0.001 |  |  | 2.70 (1.49, 4.9) | 0.001 |  |  |
| Cerebrovascular disease | 3.34 (1.75, 6.38) | <0.001 |  |  | 1.96 (0.48, 8.09) | 0.35 |  |  | 4.30 (2.07, 8.94) | <0.001 |  |  |
| Chronic liver disease | 1.73 (0.91, 3.29) | 0.10 |  |  | 1.41 (0.44, 4.55) | 0.56 |  |  | 1.92 (0.88, 4.16) | 0.10 |  |  |
| Chronic renal disease | 5.47 (1.35, 22.16) | 0.02 |  |  | 4.21 (0.03, 29.39) | 0.41 |  |  | 8.70 (2.13, 35.55) | 0.003 |  |  |
| Sarcopenia (reference: no)* | 2.36 (1.60, 3.48) | <0.001 | 1.57 (1.05, 2.35) | 0.03 | 2.61 (1.35, 5.04) | 0.004 |  |  | 2.27 (1.4, 3.68) | 0.001 |  |  |
| Fat volume index (reference: lower three quartiles) | 1.00 (0.67, 1.49) | >0.999 |  |  | 1.18 (0.62, 2.25) | 0.61 |  |  | 0.91 (0.54, 1.51) | 0.7 |  |  |
| Subcutaneous fat volume index (reference: lower three quartiles) | 0.79 (0.51, 2.21) | 0.27 |  |  | 0.84 (0.42, 1.7) | 0.63 |  |  | 0.75 (0.43, 1.3) | 0.3 |  |  |
| Visceral fat volume index (reference: lower three quartiles) | 1.53 (1.05, 2.21) | 0.03 |  |  | 1.98 (1.09, 3.58) | 0.02 | 2.08 (1.05, 4.11) | 0.04 | 1.29 (0.8, 2.07) | 0.3 |  |  |
| Subcutaneous fat attenuation (reference: lower three quartiles) | 1.74 (1.19, 2.53) | 0.004 |  |  | 1.67 (0.9, 3.1) | 0.11 |  |  | 1.78 (1.1, 2.86) | 0.02 |  |  |
| Visceral fat attenuation (reference: lower three quartiles) | 1.55 (1.07, 2.26) | 0.02 | 1.58 (1.08, 2.32) | 0.02 | 1.55 (0.84, 2.87) | 0.16 | 2.3 (1.14, 4.65) | 0.02 | 1.65 (1.03, 2.63) | 0.04 | 1.96 (1.22, 3.16) | 0.005 |
| Subcutaneous fat volume index to visceral fat volume index ratio (reference: lower three quartiles) | 0.56 (0.34, 0.90) | 0.02 |  |  | 0.45 (0.19, 1.06) | 0.07 |  |  | 0.62 (0.35, 1.11) | 0.11 |  |  |

BMI: body mass index

Multivariable Cox proportional hazard regression analysis with backward elimination was performed with variables that had *P*-values <0.2 in the univariable analysis.

The variance inflation factors (VIF) between variables in multivariable Cox regression analysis were <5

* The cutoff value for lumbar SMI was 55 cm^2^/m^2^ for men and 39 cm^2^/m^2^ for women

† Non-cancer mortality cases were excluded.

‡ Cancer mortality cases were excluded.

§ Multiple imputation was performed for smoking status.

Table S4. Log-rank test for sarcopenic obesity defined by visceral fat abnormality

| Outcome | Category | Event | *P*-values for comparison | | | |
| --- | --- | --- | --- | --- | --- | --- |
|  |  |  | Sarcopenia with visceral fat abnormality* | Sarcopenia without any visceral fat abnormality* | Non sarcopenia but with visceral fat abnormality* | Non sarcopenia without any visceral fat abnormality* |
| Overall mortality | Sarcopenia with visceral fat abnormality* | 9% (65 of 690) | N/A | <0.001 | <0.001 | <0.001 |
|  | Sarcopenia without any visceral fat abnormality* | 4% (28 of 730) | <0.001 | N/A | 0.19 | 0.55 |
|  | Non sarcopenia but with visceral fat abnormality* | 2% (15 of 661) | <0.001 | 0.19 | N/A | 0.57 |
|  | Non sarcopenia without any visceral fat abnormality* | 3% (20 of 639) | <0.001 | 0.55 | 0.57 | N/A |
| Cancer mortality | Sarcopenia with visceral fat abnormality* | 4% (26 of 653) | N/A | 0.002 | 0.001 | 0.001 |
|  | Sarcopenia without any visceral fat abnormality* | 1% (9 of 709) | 0.002 | N/A | 0.71 | 0.65 |
|  | Non sarcopenia but with visceral fat abnormality* | 1% (6 of 655) | 0.001 | 0.71 | N/A | 0.9 |
|  | Non sarcopenia without any visceral fat abnormality* | 1% (6 of 622) | 0.001 | 0.65 | 0.9 | N/A |
| Non-cancer mortality | Sarcopenia with visceral fat abnormality* | 6% (40 of 669) | N/A | <0.001 | <0.001 | <0.001 |
|  | Sarcopenia without any visceral fat abnormality* | 3% (18 of 716) | <0.001 | N/A | 0.2 | 0.79 |
|  | Non sarcopenia but with visceral fat abnormality* | 1% (9 of 658) | <0.001 | 0.2 | N/A | 0.41 |
|  | Non sarcopenia without any visceral fat abnormality* | 2% (14 of 630) | <0.001 | 0.79 | 0.41 | N/A |

N/A: not applicable

* Visceral fat abnormality included high visceral fat attenuation or visceral fat volume index, defined as the highest quartile.

Table S5. Log-rank test for sarcopenic obesity defined by the visceral fat volume index within the same body mass index category

| Category | Overall mortality |  | Cancer mortality |  | Non-cancer mortality |  |
| --- | --- | --- | --- | --- | --- | --- |
|  | Event | P-value | Event | P-value | Event | P-value |
| BMI underweight |  |  |  |  |  |  |
| Sarcopenia with high visceral fat volume index* | 0% (0 of 0) | N/A | 0% (0 of 0) | N/A | 0% (0 of 0) | N/A |
| No sarcopenia with high visceral fat volume index* | 7% (6 of 92) |  | 2% (2 of 88) |  | 4% (4 of 90) |  |
| BMI normal |  |  |  |  |  |  |
| Sarcopenia with high visceral fat volume index* | 11% (16 of 144) | <0.001 | 4% (6 of 135) | 0.01 | 7% (10 of 140) | 0.007 |
| No sarcopenia with high visceral fat volume index* | 4% (66 of 1592) |  | 1% (23 of 1548) |  | 3% (43 of 1567) |  |
| BMI overweight or obese |  |  |  |  |  |  |
| Sarcopenia with high visceral fat volume index* | 11% (16 of 148) | <0.001 | 6% (9 of 141) | <0.001 | 5% (7 of 140) | 0.05 |
| No sarcopenia with high visceral fat volume index* | 3% (24 of 744) |  | 1% (7 of 727) |  | 2% (17 of 736) |  |

BMI: body mass index; N/A: not applicable

BMI was categorized as underweight (<18.5 kg/m^2^), normal (18.5-24.9 kg/m^2^), overweight (25-29.9 kg/m^2^), and obese (>30 kg/m^2^)

* High visceral fat volume index defined as the highest quartile.
